# A Retrospective Analysis of Crayfish-Related Rhabdomyolysis (Haff Disease)

**DOI:** 10.1155/2019/4209745

**Published:** 2019-07-01

**Authors:** Changbao Huang, Liangfei Peng, Nengkai Gong, Cheng Xue, Weihua Wang, Jinghan Jiang

**Affiliations:** Department of Emergency Medicine, Yijishan Hospital, Wannan Medical College, Wuhu, Anhui 241001, China

## Abstract

**Objective:**

To investigate the epidemiologic and etiologic factors, clinical features, therapeutic regimen, and prognosis of crayfish-related rhabdomyolysis (Haff disease).

**Methods:**

Retrospectively analyzed the clinical data of 29 patients with crayfish-related rhabdomyolysis (Haff disease) from July to August 2016, summarized the clinical characteristics, and evaluated the prognosis.

**Results:**

Clinical data of a total of 29 cases of Haff disease were retrospectively analyzed. The disease onset occurred after consumption of cooked crayfish with the incubation period ranging from 1 h to 48 h. There were no gender differences and significantly elevated CK in the duration with peak value of 41575.0U/L; the median value was 2445.0U/L (range: from 1187.0 U/L to 4722.0 U/L) and there was coincident elevated CK-MB. There was also no hepatorenal damage and transient urinalysis was abnormal. The most common presenting symptoms were myalgia (100%), weakness and numbness (51.7%), chest tightness and chest pain (41.4%), back pain (41.4%), and extremities pain (37.9%). All the patients recovered and no patients died.

**Conclusions:**

Crayfish-related rhabdomyolysis (Haff disease) is a kind of a case or cluster of patients present with severe myalgia or weakness of unknown etiology and mechanism disease; however, the clinical signs and symptoms are relatively mild with favorable outcome.

## 1. Introduction

Rhabdomyolysis (RM) is a group of clinical syndromes, which is characterized by myalgia, myasthenia, muscle swelling, stiffness, and tea-color urine [1]. The cell membrane integrity of striated muscle is impaired by traumatic or nontraumatic factors and, consequently, led to the release of active components in cells to blood circulation; then a series of clinical symptoms appeared [2]. About 80% of the patients are induced by nontraumatic factors [3], among which drugs are the main pathogenic factors. Although the incidence of RM caused by food source factors is low, RM caused by eating aquatic products has been reported since 1924 [4]. Haff disease is a clinical syndrome characterized by myalgia and other symptoms within 24 hours after the consumption of fish or shrimp, often accompanied by myoglobinuria [5]. In the United States, only 29 cases have been reported so far [6]. In China, Yuan et al. [7] first reported 6 cases of Haff disease in 2001. However, Diaz et al. [6] searched literatures and found that there were only 54 cases of Haff disease in China. Therefore, this disease is still rare in China, which leads to the possibility of delayed diagnosis in clinical practice. This paper reports a total of 29 cases with crayfish-related rhabdomyolysis (Haff disease), clarifies the epidemiological characteristics and clinical features in China, and improves the level of clinical diagnosis and treatment.

## 2. Materials and Methods

Patients who were admitted to Emergency Department of Yijishan Hospital of Wannan Medical College from July to August 2016 were enrolled. All the data were retrospectively analyzed and all patients had a clear history of eating crayfish and had onset symptoms of rhabdomyolytic syndrome within 24 hours after eating crayfish. Baseline demographics such as age, gender, onset of symptoms, time to initial symptoms, time to admission, oral amount of crayfish, and length of stay were collected, and blood routine, biochemical, coagulation function, and arterial blood gas analyses were tested.

Those with a positive ingestion crayfish or other history of aquatic products within 24 hours, with markedly elevated serum creatine phosphokinase (fivefold or more rise over normal value, namely, > 1000U/L), and with CK isoenzyme type MB (CK-MB) less than 5% of CK [8] were enrolled; meanwhile, rhabdomyolysis caused by other definite reasons such as trauma and drugs was excluded. After admission, all patients received biochemical examination to confirm the diagnosis and evaluate the condition and renal function.

All patients received adequate fluid resuscitation with 0.9% sodium chloride injection (no potassium and lactic containing solutions); the infusion speed began with a rate of 1.5 L/h to maintain urine output about 200-300 ml/h, corrected the electrolyte and metabolic abnormalities, alkalization of the urine with sodium bicarbonate continuous infusion to prevent renal tubular jams, protection of gastric mucosa to reduce the stress ulcer, careful monitoring of cardiopulmonary function, biochemistry data, routine urine, and arterial blood gas analysis; blood purification should be performed with critical cases [2].

Data were expressed as median (25th quantile, 75th quantile) for continuous variables and number (percentage) for categorical variables. All statistical analyses were performed with SPSS version 20.0 software and all graphs were made by GraphPad Prism version 5.0 software.

## 3. Results

A total of twenty-nine cases presented with rhabdomyolysis in East China between July and August 2016; all cases reported ingestion of crayfish within 24 hours before onset of symptoms and occurred in small clusters; there were no outbreaks in East China during those months. There was no difference in morbidity between the genders, and the median age was 39.5 years (range from 21 to 66 years), and median quantity ingested of the crayfish was 225 grams (range from 50 to 2500 grams). The median onset time was 8 hours (range from 1 to 48 hours) after consumption of crayfish; of all cases, only 10 patients needed to be hospitalized; the rest were treated in the emergency room, and the median length of stay was 4 days (range from 1 day to 9 days). All patients had a good prognosis after follow-up, and no deaths occurred (Table 1).

All cases had rapid onset of myalgia, followed by the inability to feel numbness (51.7%), chest pain (41.4%), back ache (41.4%), and limbs pain (37.9%), respectively. Only four patients had brown urine/hematuria (13.8%) (Table 2).

**Figure 1:**
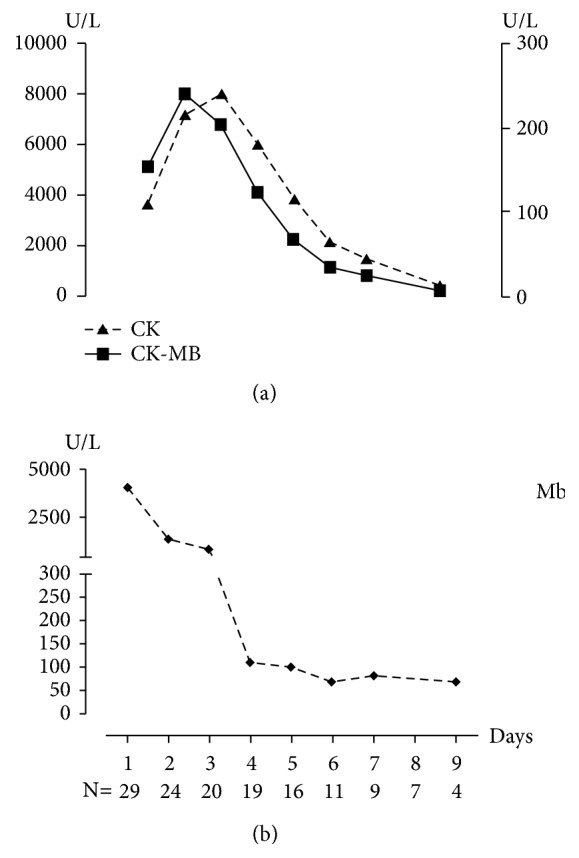
The trend graph of CK, CK-MB, and Mb in cases with rhabdomyolysis. (a) The trend graph of CK and CK-MB during hospitalization. (b) The trend graph of Mb during hospitalization.

The predominant laboratory abnormality was elevated CK and CK-MB; the CK was elevated to the maximum level of 41,575.0 U/L with a median of 2,445.0 U/L (1187.0 to 4722.0 U/L), and it reached the peak level at 2nd to 3rd day after admission with a median of 4,815.0U/L; meanwhile, the CK-MB was also significantly increased with a median of 110.0 U/L (72.5 to 155.0 U/L) and a maximum of 4815.0U/L; its elevated trend was consistent with the CK's. The MB fraction was 2%~8% with mean of 4.4%. Myoglobin (Mb) was also detected among a total of 28 cases; the median value was 1020.0 U/L (237.6~6397.3) which was significantly elevated; the Mb reached the peak value on the admission (Figure 1). All patients had normal liver and kidney function on admission. Urine routine examination was performed in twenty-two cases; urine erythrocytes were positive in only nine cases, +~+++, while urine protein was positive in thirteen cases, +~+++ (Table 3).

## 4. Discussion

Since the first report of Haff disease in 1924, there have been several sporadically or in small clusters epidemics; among them, the number of cases of Haff disease in eastern Europe and Russia is much more than that in other countries, which exceeds more than 1000 cases [8]. In recent decades, there were only some scattered reports in Asia and America, and, in China, there were only two outbreaks which involved a large number of patients who had been reported [9–11]. Therefore, Haff disease is a disease with low prevalence that can easily be misdiagnosed and undiagnosed. Haff disease is a syndrome of rhabdomyolysis characterized by muscle pain and muscle weakness with or without brown urine/hematuria [5]. Laboratory examination shows that CK will rapidly rise to a fivefold or greater elevation after onset of symptoms, accompanied with elevated CK-MB; however, the MB fraction is less than 5%, without pyrexia, liver, and kidney function damaged. From 1997 to 2014, nine of the twenty-seven patients with rhabdomyolysis in the United States were diagnosed with Haff disease because of eating crayfish [8]; all of them were characterized by muscle pain (100%), muscle weakness (100%), chest pain and tightness (100%), nausea and vomiting (100%), and sweating (100%), respectively, without the symptoms such as dizziness headache, abdominal pain, and diarrhea. Our results may be different from the previous studies. In our study, we found that the predominant performances of Haff disease were muscle pain (100%), followed by muscle weakness numbness (51.7%), chest pain and tightness (41.4%), back pain (41.4%), and limb pain (37.9%), respectively. However, there was no brown urine/hematuria. Meanwhile, we also found that levels of CK, CK-MB, and Mb were rapidly elevated at the onset except that seven of twenty-nine cases' initial CK did not reach the standard of five times normal at admission which significantly increased after 24 hours. In some cases, CK does not rise to fivefold or more than normal until 12 hours after muscle is injured; therefore, we should detect CK repeatedly. In this study, we found that CK and CK-MB reached the peak level on the second to third day after admission and then quickly recovered to normal, while the Mb reached peak level at onset then a downward trend; those results were consistent with reports by Buchholz et al. [5].

The red swamp crayfish, also named* Procambarus clarkii *(*P. clarkii*), is the most common farm-raised crayfish in China, especially in the middle and lower reaches of the Yangtze River Valley because the conditions of the areas are favorable for the growth and reproduction. Crayfish are bottom-feeder freshwater animals, which feed on algae [12]. In China, almost all outbreaks resembling Haff disease were reported due to eating crayfish except one outbreak of Haff disease in Guangdong province in 2009 which was caused by freshwater pomfret [10]. In particular, in recent years, more and more Chinese people prefer to eat crayfish during the whole summer and fall every year, which leads to the onset of Haff disease concentrated in this period [13–15]. However, to date, the pathogenesis of crayfish-related rhabdomyolysis has not yet been identified. Direct or indirect injury to cell membrane can cause pump dysfunction (Na/K-ATPase, Ca^2+^ ATPase pump), which leads to elevated intracellular Na^+^ and Ca^2+^ concentration. High intracellular calcium levels enhance the activation of calcium-dependent proteases and phospholipases, which cause the destruction of myofibrillar, cytoskeletal, and membrane proteins and ultimately lead to rhabdomyolysis syndrome [2, 16]. Meanwhile, it is also not clear what is the etiological of crayfish-related rhabdomyolysis. Some unknown hexane-soluble products extracted from boiled crayfish can lead to striated muscle injury and red-brown urine in animal experiments [5, 17, 18]; therefore, it is thought that the heat-stable toxins in crayfish may be the cause of Haff disease [8, 11]. Other scholars presumed that the etiologies may include arsenic [5] or palytoxin [17] because of absence of fever symptom; nevertheless, tests of the crayfish for toxins were either negative or below toxicity thresholds [5], and the same results appeared in Liang's study [19].

Although marine or freshwater fish-associated rhabdomyolysis had been reported in many literatures with a few serious cases suffering residual effects of weakness or cognitive dysfunction and even death [8, 20, 21], to date, there had been no dying cases in China, and the clinical symptoms of all reported cases were relatively mild. There are not any antitoxins for Haff disease, and treatment is entirely supportive [8], such as intravenous fluid hydration, intravenous sodium bicarbonate, intravenous loop (furosemide) or osmotic (mannitol) diuretics, or extracorporeal renal replacement techniques, including hemofiltration and hemodialysis [22]. Remarkably, the initial clinical presentation of some cases may mimic myocardial infarction, so that we must exclude myocardial infarction by electrocardiograms and serum troponin (troponin I and T) levels, especially those with CK-MB over 5%. Xie et al. [23] concluded that diagnosis of Haff disease should be based on presenting clinical and laboratory features and can be further supported by muscle biopsy and bone scintigraphy. In the meantime, the severity of the disease may be independent of oral amount [24], with the exception of freshwater-pomfret-induced rhabdomyolysis [10]. In our study, most of the patients took more than 500g of crayfish; more importantly, those patients had eaten the same crayfish with their friends free of this disease; therefore, we believe that the consumption amount is not related to the onset, and it may be related to the specific constitution of the patients.

In conclusion, the primary pathogenic factor of Haff disease in China is crayfish. The confirmed diagnosis of Haff disease should be based on initial presenting signs and symptoms and laboratory tests. This retrospective study analyzed the epidemiological and clinical characteristics of Haff disease to aim to help clinicians further recognize this disease. Of course, this study also has some limits; we have not detected the toxins yet. More toxicological investigations in larger case clusters are recommended to identify the toxin responsible for Haff disease.

## Figures and Tables

**Table 1:** Baseline demographics of twenty-nine cases of rhabdomyolysis.

	n=29
Male	15/29
Female	14/29
Age (years)	39.5±10.8
Time to onset symptoms (hours)	10.3±10.3
Time to admission (hours)	16.8±14.4
Oral crayfish amount (grams, median, IQR)	500 (225-1000)
Length of stay (days, median, IQR)	4.0 (2.5-6.0)

**Table 2:** Symptoms of all total of 29 rhabdomyolysis cases (N, %).

	N	Percent (%)
Myalgia	29	100.0%
Back pain	12	41.4%
Waist pain	9	31.0%
Limbs pain	11	37.9%
Muscle weakness or numbness	15	51.7%
Nausea or vomiting	6	20.7%
Dizziness	1	3.4%
Chest pain or chest congestion	12	41.4%
Abdominal pain or bloating	7	24.1%
Diarrhea	1	3.4%
Profuse sweating	1	3.4%
Shortness of breath	2	6.9%
Brown urine/hematuria	4	13.8%

**Table 3:** Laboratory test results of twenty-nine cases during hospitalization.

	N	median, IQR or mean value	Reference value
*Serological examination*			
Initial CK (U/L)	29	2445.0 (1187.0~4722.0)	55~170
Peak CK (U/L)	29	4815.0 (2466.0~88711.5)	55~170
Initial CK-MB (U/L)	29	110.0 (72.5~155.0)	<16
Peak CK-MB (U/L)	29	130.0 (85.0~250.0)	<16
The MB fraction (%)		4.4±1.8	
Initial LDH (U/L)	29	594.0 (535.0~664.5)	313~618
Peak LDH (U/L)	29	700.0 (577.0~1044.0)	313~618
Initial AST (U/L)	29	86.0 (53.0~113.0)	15~46
Peak AST (U/L)	29	106.0 (67.0~247.0)	15~46
Initial Mb (ng/ml)	28	1020.0 (237.6~6397.3)	11.6~73
Peak Mb (ng/ml)	28	1221.0 (397.0~6471.0)	11.6~73
*Renal Function*			
Initial Cre (umol/L)	29	62.9±12.6	46~92
Initial BUN (mmol/L)	29	8.4±13.1	2.5~6.1
*Liver Function*			
Initial ALT (U/L)	28	53.3±31.5	13~69
Initial ALP (U/L)	24	76.2±19.7	38~126
Initial TBiL (umol/L)	23	12.7±5.4	3~22
*Urine screening*			
Initial urine protein	22	9/29	negative
Initial urine erythrocytes	22	13/29	negative

## Data Availability

No data were used to support this study.
